# Lamellar-like Electrospun Mesoporous Ti-Al-O Nanofibers

**DOI:** 10.3390/ma12020252

**Published:** 2019-01-14

**Authors:** Oren Elishav, Liz Poliak, Iris Naamat, Vadim Beilin, Gennady E. Shter, Gideon S. Grader

**Affiliations:** 1 The Nancy and Stephen Grand Technion Energy Program, Technion—Israel Institute of Technology, Haifa 3200003, Israel; orene@technion.ac.il; 2 The Wolfson Department of Chemical Engineering, Technion – Israel Institute of Technology, Haifa 3200003, Israel; liz.poliak333@gmail.com (L.P.); iris.n91@gmail.com (I.N.); vadimbe@technion.ac.il (V.B.); shter@technion.ac.il (G.E.S.)

**Keywords:** electrospinning, nanofibers, ceramic materials, porous

## Abstract

Ceramic oxides nanofibers are promising materials as catalysts, electrodes and functional materials. In this report, a unique lamellar-like mesoporous structure was realized for the first time in a new system based on titania and alumina. The final structure was found to be highly dependent on the process conditions which are outlined herein. In view of the similar architecture we recently obtained with Fe-Al-O fibers, the pore formation mechanism we outline herein is general and is applicable to additional systems.

## 1. Introduction

Nanofibers with complex architectures of ceramic oxides are promising materials for various applications such as catalysis, battery electrodes, nano-electronics, sensors and more [1]. Therefore, it is necessary to develop general yet simple methods to effectively synthesize such structures from different materials. In recent years, Electrospinning (ES) has become a promising and effective method to produce nanofibers with designer maker complex porous structures with advantageous properties and large surface area [2,3,4].

Titanium dioxide (TiO_2_) is used for many applications such as photocatalysis and electrodes [5,6,7]. Electrospinning can serve as the method of choice to prepare TiO_2_ for these applications [8]. Previous research has indicated that the addition of aluminum can improve TiO_2_ photocatalytic performance, reduce recombination of photogenerated electrons [9,10], and enhance the performance of TiO_2_-based lithium battery anodes [11]. Therefore, significant improvement in device performance is expected via nanofibers architecture refinement and by the incorporation of additives. Furthermore, mesoporous TiO_2_ nanofibers have been shown to have superior photoelectrode [12] properties relative to nano-powders and standard nonporous nanofibers.

Recently, novel nanofibers with unique lamellar-like mesoporous structure were produced for heterogeneous catalysis application [13]. The precursor solution included PVP, Fe(AcAc)_3_ and Al(AcAc)_3_. During thermal treatment of these fibers, phase separation or segregation occurs within them, consisting of metals organic liquid inclusions due to a low melting point of one or more of the metal precursors within the nanofibers. The molten inclusions are squeezed into lamellar like structures due to the polymer’s shrinkage. The suggested formation mechanism implied that this unique structure could be obtained in other systems. In this study, we demonstrate for the first time the formation of lamellar-like mesoporous structure in a new Ti-based system. The role of the precursor composition is outlined. These Ti-based nanofibers have promising properties for catalysis and other applications.

## 2. Materials and Methods

Precursor preparation included mixing of three separate solutions: polyvinylpyrrolidone (PVP) (MW 1,300,000 g/mol, Aldrich, St. Louis, MO, USA) in absolute ethanol (Carlo Erba, Val de Reuil, France); Titanium(IV) isopropoxide (TTIP) (Strem Chemicals, Inc., Newburyport, MA, USA) in Acetylacetone (AcAc) (Aldrich, St. Louis, MO, USA); Al(AcAc)_3_ (Strem Chemicals, Inc., Newburyport, MA, USA) in glacial acetic acid (AA) (Frutarom, Herzliya, Israel). The composition of these solutions was altered, as shown in Table 1.

The ES system consists of high voltage supply (SL40P60, Spellman, Hauppauge, NY, USA), with grounded aluminum vertically rotating disc-collector, a syringe pump (KDS100, KD Scientific, Holliston, MA, USA) and an injection needle. The applied voltage was 25 kV with a tip to collector distance (TCD) of 15 cm. The relative humidity inside the system container is in the range of 45–55%. After electrospinning, the resulting “green” fiber mats were vacuum dried at 40 °C for 12 h to remove residual solvents. The fibrous mat’s phases were identified by X-ray diffraction (Rikagu SmartLab 9 kW).

The samples were characterized by HRSEM/EDS, thermal analysis coupled with evolved gas analysis (MS), XRD and N_2_ adsorption-desorption. Samples of ~40 mg in 100 µL alumina crucibles were analyzed by simultaneous thermal gravimetric and differential thermal analyses (TGA/DTA) at ambient pressure (Setsys Evolution 1750, Setaram, Caluire, France). The samples were heated from 25 to 1100 °C at 5 °C∙min^−1^ under air flow of 20 ml∙min^−1^. The obtained data was treated using the Calisto Processing software (AKTS and Setaram). Morphology was investigated by HRSEM (ULTRA plus; Zeiss, Zurich, Switzerland).

## 3. Results

**Figure 1:**
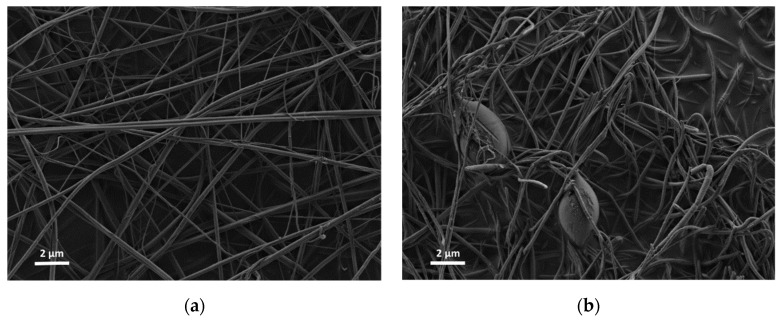
Electrospun fibers before thermal treatment (**a**) of Solution II, similar results obtained with solutions III and IV (**b**) of solution I showing beads formation.

**Figure 2:**
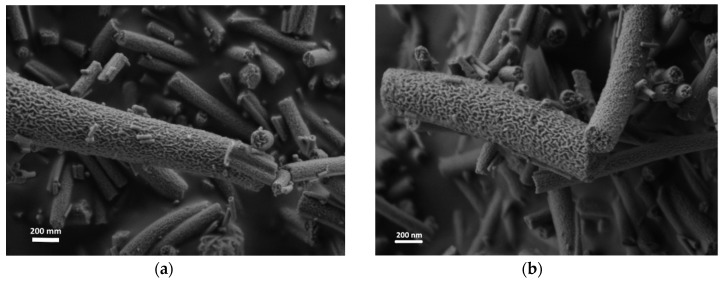

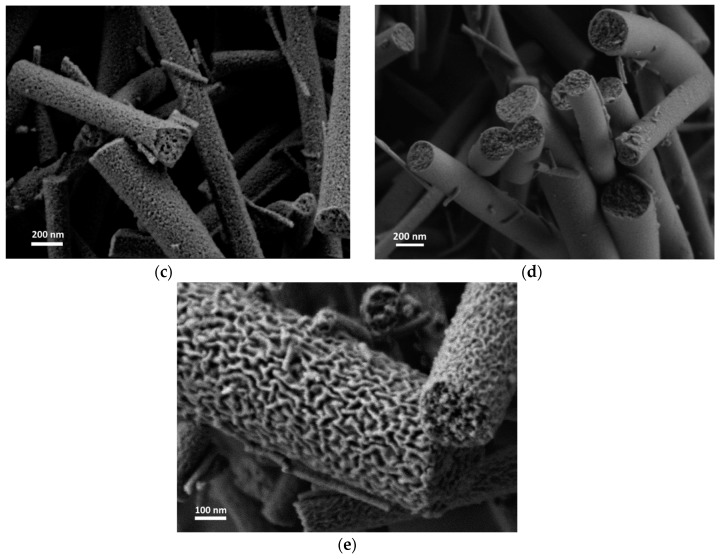
Electrospun nanofiber after thermal treatment (**a**) solution I (**b**) solution II (**c**) solution III (**d**) solution IV (**e**) Solution II, higher magnification.

**Figure 3:**
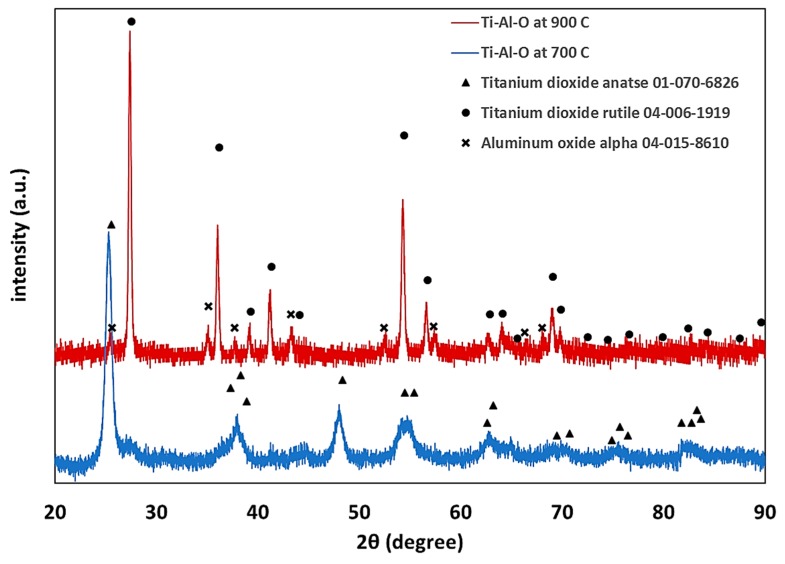
An XRD pattern of sintered nanofibers solution II.

**Figure 4:**
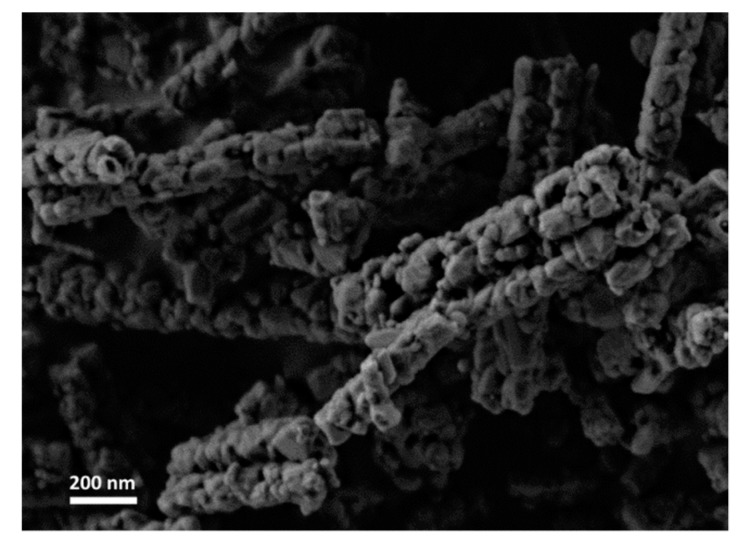
HRSEM image of Ti-Al-O nanofiber after sintering to 900 °C (Solution II).

Nanofibers were prepared using a single nozzle ES system with composition shown in Table 1. All the fiber components are uniformly distributed in the ES precursor solution (II-IV), as well as in the dried fibers prior to thermal treatment, as shown in Figure 1a. At a low PVP content (Solution I), few scattered beads are formed (Figure 1b) [14]. Further decrease of the PVP content resulted in an unspinnable precursor. Before thermal treatment, the fibers had a bimodal distribution consisting of fibers with a large diameter of 600–1000 nm (average 672 nm, ±206 nm) and fibers with a smaller diameter in the 100–170 nm range (average 138 nm, ±22 nm). The dried ‘green’ fibers were heated in a multi-step heating profile up to 700 °C (Figure S1). Different final morphologies were obtained by varying the PVP content relative to the other precursor components (Figure 2). As expected, the fibers diameter shrunk to less than half the initial (green) diameter during thermal treatment, so after heating they had a bimodal distribution consisting of fibers with a large diameter, i.e., 200–400 nm (average 292 nm, ±30 nm), and fibers with a smaller diameter, i.e., in the 40–80 nm range (average 63 nm, ±13 nm). Thus, after thermal treatment, a 55% shrinkage in diameter was observed. This shrinkage is consistent with earlier findings in our work with other ceramic-polymer systems [15,16]. The lamellar-like pores appear after sintering in solution I and II. Single point BET calculations showed that the surface area of lamellar-like structure was in the range of 150–170 m^2^∙gr^−1^. The pore size in the fibers obtained by BJH method was 4–5 nm. Increasing the PVP composition (Solution II) resulted in stable ES operation without the formation of beads. After sintering, the fibers display lamellar like pores (Figure 2b). Increasing the polymer content further (Solution III) results in the formation pores but with different structures (Figure 2c). Since the PVP content is increased relative to the ceramic precursor (Ti- and Al-based components), the segregation or phase separation during the thermal treatment is less significant, and only small separated inclusions of liquid are formed. At high PVP content (Solution IV), the obtained fibers have a relatively smooth surface (Figure 2d). EDS was used to verify the content of Ti and Al in the fibers (Figure S2); the carbon signal is due to the presence of carbon tape as the sample adhesive layer. In addition, the X-ray diffraction (XRD) pattern only shows anatase titanium dioxide phase; the alumina phase is not observed (Figure 3). Therefore, either the aluminum oxide is amorphous or the crystalline alumina phase signals peaks are below the detection level. Increasing the final temperature from 700 °C to 900 °C results in the destruction of the lamellar-like structure (Figure 4) due to growth of the ceramic particles. As expected, at 900 °C, the TiO_2_ phase changed from anatase to rutile with TiO_2_ [17], and the α-Al_2_O_3_ phase is also observed (Figure 3).

**Figure 5:**
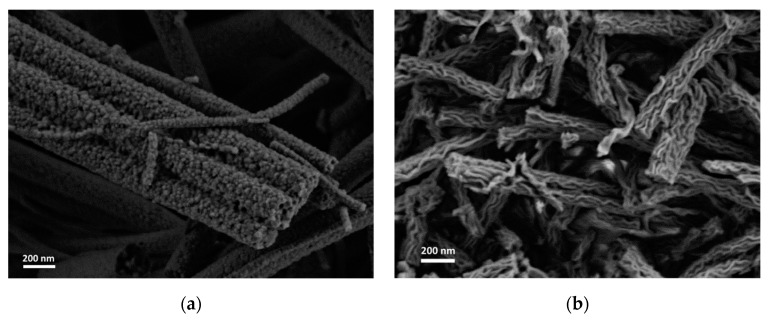
HRSEM images of electrospun and thermally treated nanofibers. (**a**) pure Titanium(IV) isopropoxide precursor, (**b**) pure Al(AcAc)_3_ precursor.

In agreement with the mechanism suggested earlier for the formation of lamellar-like nanofibers, the presence of an aluminum-organic component with a low melting point and high volatility facilitated the structure formation [13]. Therefore, pure TiO_2_ with the same PVP-to-ceramic precursor ratio as that of solution II showed different morphology (Figure 5a). In contrast, a pure Al-based solution with the same PVP-to-ceramic precrusor ratio as that of solution II showed the same lamellar-like nanofiber morphology (Figure 5b).

**Figure 6:**
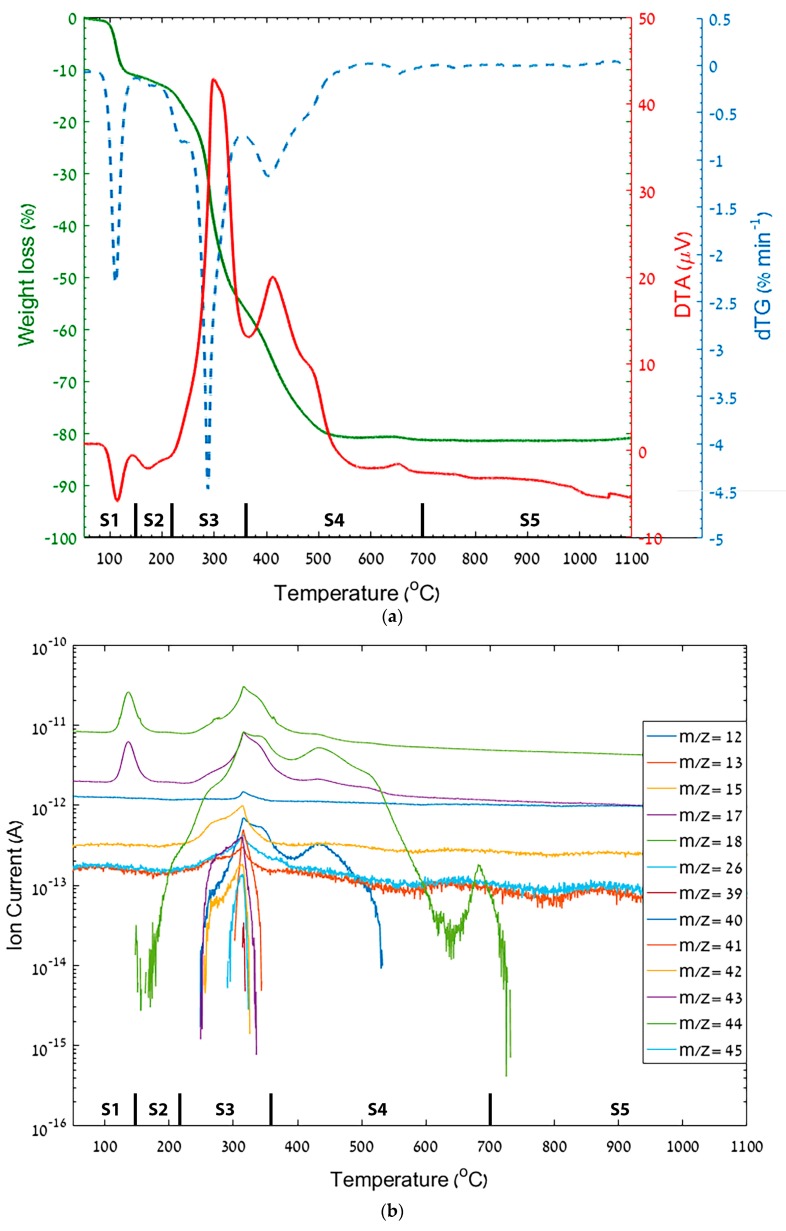
Thermal analysis with evolved gas analysis of Ti-Al-O nanofibrous mat. Vertical dashed lines represent a process stage (denoted as S1–S5). (**a**) TGA/DTA/DTG curves. (**b**) Evolved gases detected by MS.

The fibrous mat was analyzed in simultaneous TGA/DTA-MS in air (Figure 6). In the first stage (S1), residual absorbed water is evaporated at 85–150 °C, as indicated by an endothermic peak with weight loss of 11.16% and MS signals (m/z = 18, 17). The second stage (S2) in the temperature range of 150–220 °C has negligible weight loss (less than 2%). At this stage, following the polymer glass transition, the fibers shrink [14], while Al(AcAc)_3_ melts and can segregate with the TTIP in inclusions of a liquid phase (i.e. phase separation). During heating, the polymer relaxes, forming non-uniform deformation of the segregated inclusions while supporting the structure and the final morphology. The next step (S3) includes PVP and metal organic part partial decomposition as indicated by a high exothermic peak with H_2_O, CO, CO_2_ and nitrogen-based compounds (NH_3_, NO_x_) signals. A second exothermic peak results from the decomposition of heavy hydrogen-poor organic parts (S4). Above 700 °C, the final morphology is achieved after full organic burnout and densification (S5).

## 4. Discussion

This paper demonstrates for the first time a unique lamellar-like mesoporous structure in Ti-based electrospun ceramic nanofibers. The structure is obtained by carful design of the precursor composition and thermal treatment at 700 °C of the fibers before thermal treatment yielding TiO_2_ in the anatase phase. By altering the PVP composition, different morphologies are obtained. At a thermal treatment final temperature of 900 °C, the fiber’s structure is destroyed and the TiO_2_ rutile phase along with alpha-alumina phase appear. Therefore, careful design of the precursor composition and thermal treatment is required to obtain the desired morphology. The unique structure we obtained consists of accessible open pores that can be promising for electrode and heterogeneous catalyst applications. Based on earlier lamellar-like structures obtained with a different system (Fe-Al-O), the results obtained herein imply that the unique pore formation mechanism we report is general and can be applied to other systems.

## Figures and Tables

**Table 1:** Electrospuns precursors’ composition.

Composition (% wt.)	Solution I	Solution II	Solution III	Solution IV
TTIP	9%	9%	8%	8%
AcAc	6%	6%	6%	6%
PVP	8%	9%	10%	12%
Ethanol	21%	21%	21%	20%
Al(AcAc)_3_	4%	4%	4%	4%
AA	53%	52%	51%	50%

## Supplementary Materials

The following are available online at http://www.mdpi.com/1996-1944/12/2/252/s1, Figure S1: Thermal treatment heating profile, Figure S2: Energy dispersive X-ray spectroscopy (EDS) for electrospun nanofibers (solution II).
