# Supplementary material for: Lamellar-like Electrospun Mesoporous Ti-Al-O Nanofibers
Source: Materials (Basel). 2019 Jan 14;12(2):252. doi: 10.3390/ma12020252 (PMC6356458; doi:10.3390/ma12020252)
Supplement: Supplementary file 1 [file materials-12-00252-s001.pdf]

materials

Communication

Supplementary information: Lamellar-like Electrospun Mesoporous Ti-Al-O Nanofibers

Oren Elishav [1], Liz Poliak [2], Iris Naamat [2], Vadim Beilin [2], Gennady E. Shter [2] and Gideon S. Grader [2,*].

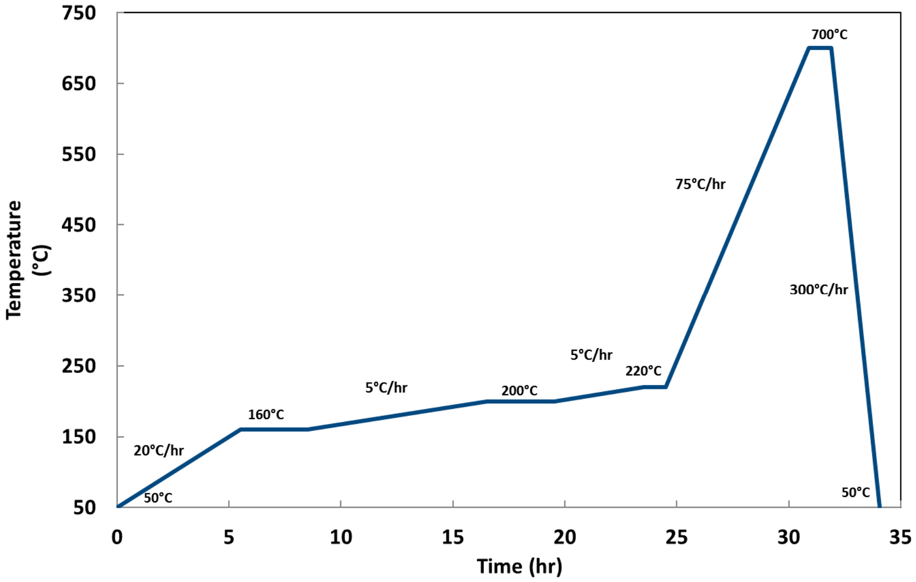

Figure S1. Thermal treatment heating profile

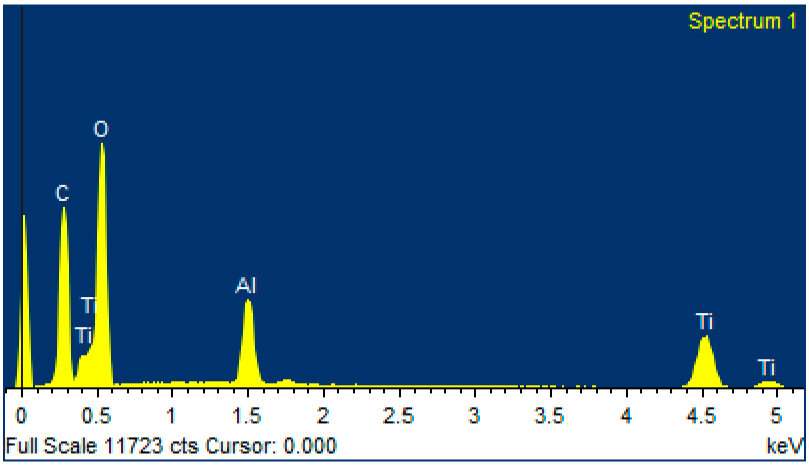

Figure S2. Energy dispersive X-ray spectroscopy (EDS) for electrospun nanofibers (solution II)

© 2018 by the authors. Submitted for possible open access publication under the terms and conditions of the Creative Commons Attribution (CC BY) license (http://creativecommons.org/licenses/by/4.0/).
